# Anterior hip dislocation with ipsilateral displaced fracture neck of femur treated by open reduction and internal fixation: case report and review of the literature

**DOI:** 10.1007/s11751-017-0292-8

**Published:** 2017-08-07

**Authors:** Ayman Mohammad El Masry

**Affiliations:** Orthopaedic Surgery Department, Faculty of Medicine, Demerdash Hospital, Ain Shams University, Abbassia Square, Cairo, 11566 Egypt

**Keywords:** Neck, Femur, Fracture, Hip, Dislocation

## Abstract

Combined fracture neck femur with anterior dislocation of the head of femur is a very rare injury with little resources available in the literature. This case report describes a case that was treated by open reduction and internal fixation with 2-year follow-up showing good radiological and functional outcome. Functional evaluation was done according to Merle d’Aubigné score modified by Matta JM, which takes into account the presence of pain, ability to walk and joint range of motion. The measured functional score was 15 points out of a maximum of 18, signifying a good postoperative result. Early intervention (within 6 h) achieving stable congruent reduction and rigid internal fixation provides the best chances for such patients.

## Introduction

The anatomical features of the hip joint confer a high degree of stability which is why dislocations of the hip occur after high-energy trauma, such as road traffic accidents, industrial accidents, sport injuries (e.g., soccer, rugby, and wrestling) or falls from a height [1]. There may be associated acetabular fractures or fractures of the head, neck or shaft of femur. Posterior hip dislocation is approximately nine times more frequent than the anterior type [2, 3].

A traumatic hip dislocation with an associated femoral neck fracture in the absence of an acetabular fracture is a rare and challenging injury. These high-energy injuries in young patients may result in significant morbidity and loss of function consequent to avascular necrosis and secondary osteoarthritis [4, 5].

The most important factor creating the anterior dislocation of the hip is forcible abduction where, in this position, the neck or trochanter impinges on the rim of the acetabulum and forces the femoral head forward through the anterior capsule. If the hip is in a concomitant position of flexion, an obturator-type dislocation may occur. If the hip is extended, a pubic type results [6]. If the force is not dissipated, then it causes a complete break in the continuity of the neck [7].

These cases are associated usually with significant trauma, and most patients have other concomitant injuries, especially to the lower extremities [7, 8].

Surgical treatment of these injuries will involve either fixation or prosthetic replacement, and with the limited number of cases described there remains little in the literature to support a definitive treatment option [9].

## Case

A 28-year-old male manual worker presented to emergency room after a car accident with severe pain in his left shoulder and left hip. Clinical examination revealed gross swelling of the left shoulder, crepitus and a prominent deformity. The left lower limb was shortened, externally rotated and the patient unable to straight leg raise. There was severe pain in the groin area with a palpable lump. Popliteal and pedal pulses were palpable.

**Fig. 1:**
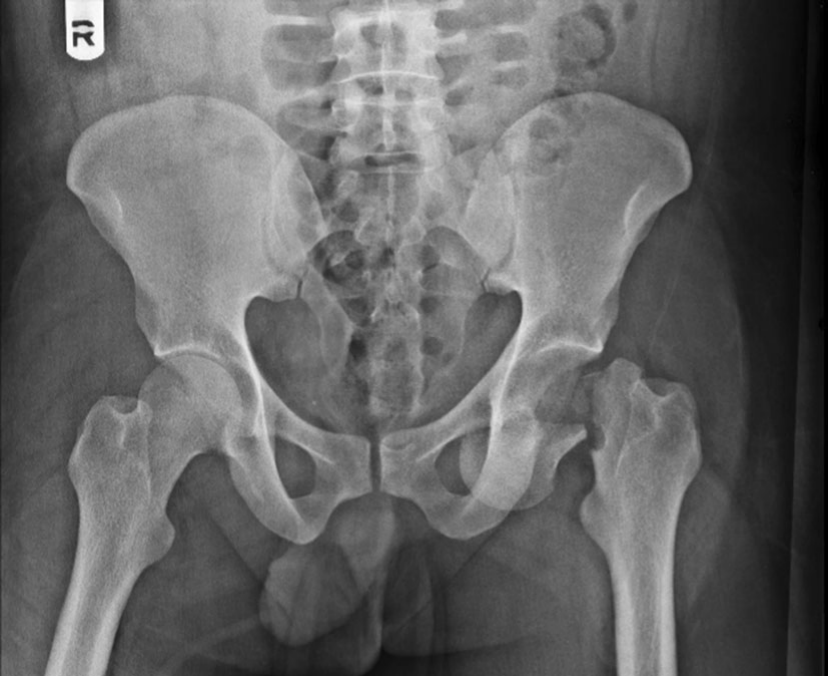
X-ray pelvis AP showing fracture neck femur with obturator-type anterior dislocation

The X-rays revealed a fracture of the left clavicle, a comminuted fracture of the left scapula and a fracture of the neck of the left femur with anterior dislocation of the femoral head (obturator type) (Fig. 1).

**Fig. 2:**
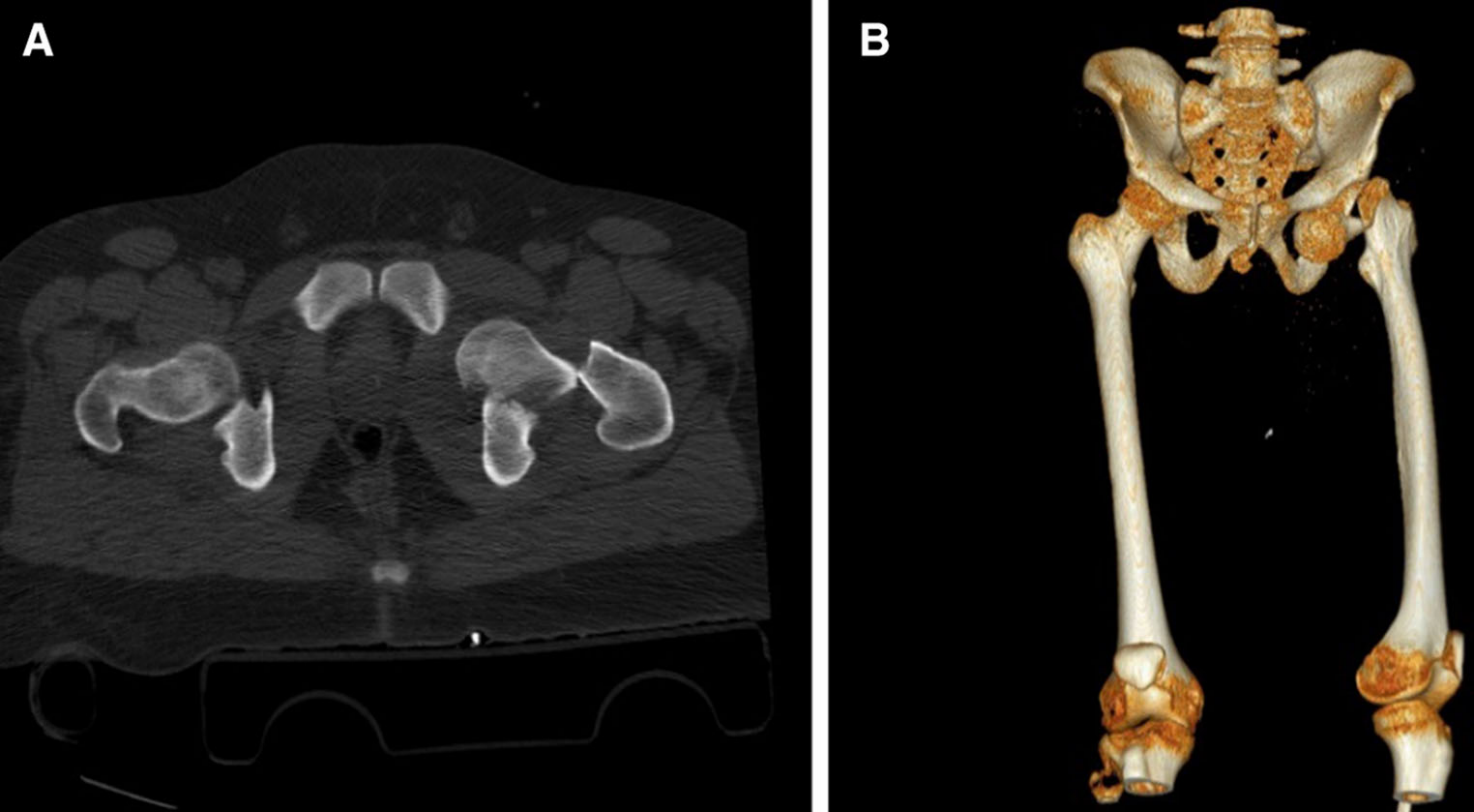
CT pelvis (**a**) with 3D reformat (**b**)

A CT scan for the hip was performed to locate the femoral head precisely and to exclude associated acetabular fractures (Fig. 2).

**Fig. 3:**
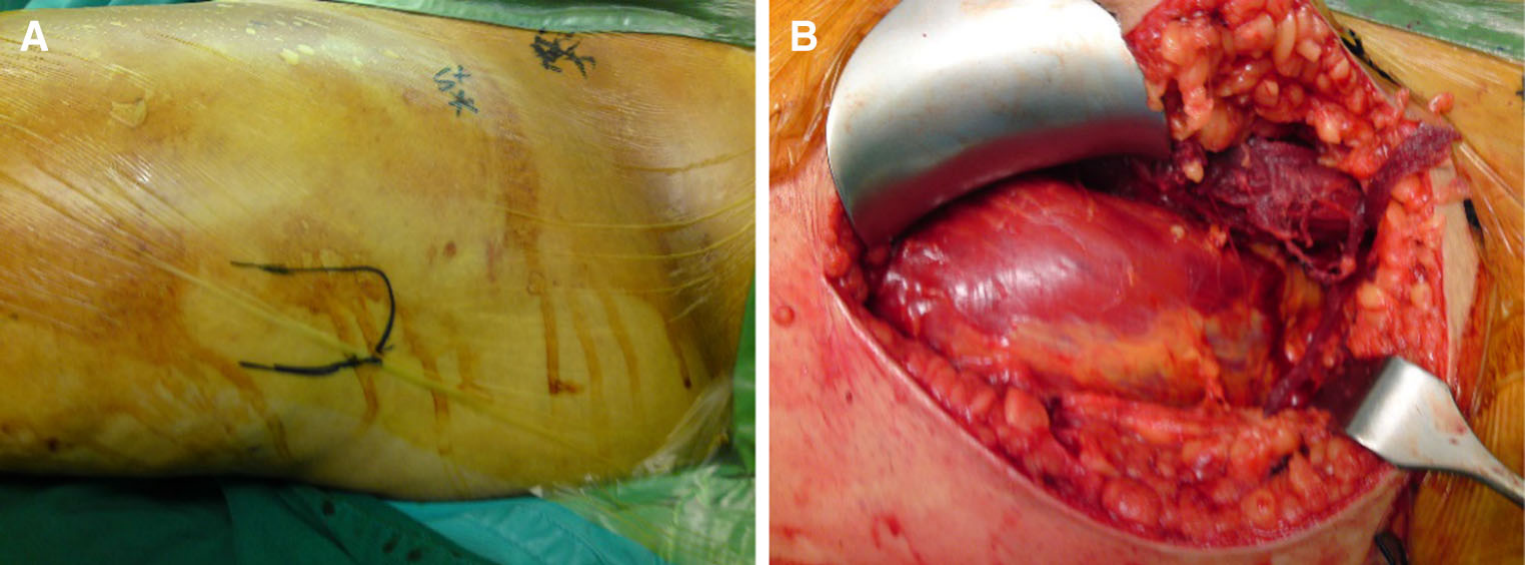
Intraoperative photographs: **a** Skin land marks for the Watson Jones incision. **b** Muscle dissection. **c** Head of femur retrieval

Open reduction and internal fixation for the fracture dislocation were arranged within 5 h after admission. Under general anesthesia and in the supine position, an anterior approach of the hip (Watson Jones approach) was utilized. Retrieval of the head was attempted under guidance of the image intensifier and, after head retrieval, the fracture pattern recognized as Pauwel’s classification type 3 with posterior comminution. The head was reduced into the acetabulum, followed by fixation by using cannulated partially threaded titanium lag screws (Fig. 3).

**Fig. 4:**
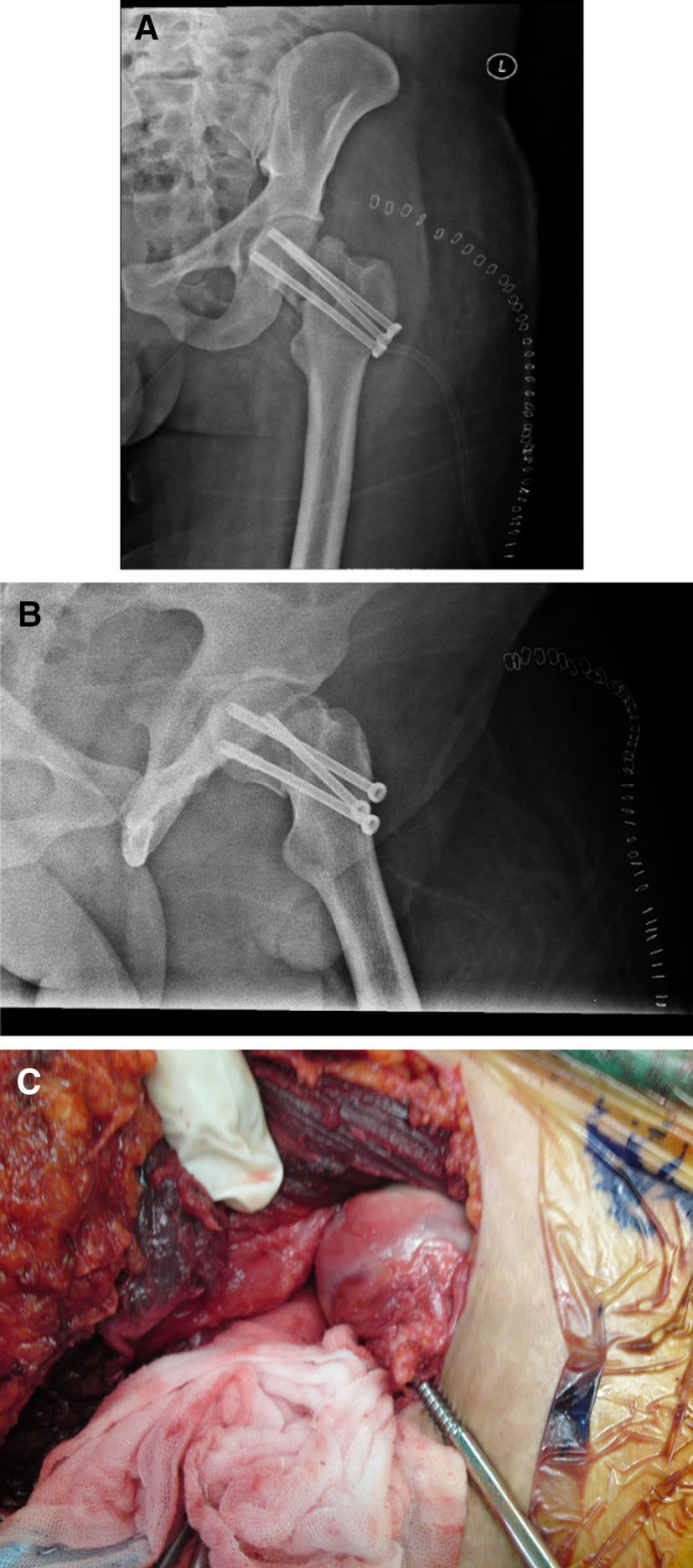
Postoperative X-rays: **a** Hip A/P view. **b** Hip lateral VIEW

Postoperative X-rays were judged satisfactory with the patient who was vitals stable and with an intact peripheral circulation (Fig. 4). A week later, open reduction and internal fixation of the clavicle fracture were performed.

The patient was discharged a week after with medications including clexane (Sanofi-Aventis) and indomethacin (to guard against heterotrophic ossification) for 6 weeks. Follow-up was carried out every 2 weeks and the patient instructed to be nonweight-bearing with static quadriceps and hamstring strengthening exercises and no straight leg raising allowed.

Six weeks after surgery, some evidence of healing was identified on X-rays, so partial weight-bearing was allowed; at 10 weeks, full weight-bearing was commenced.

**Fig. 5:**
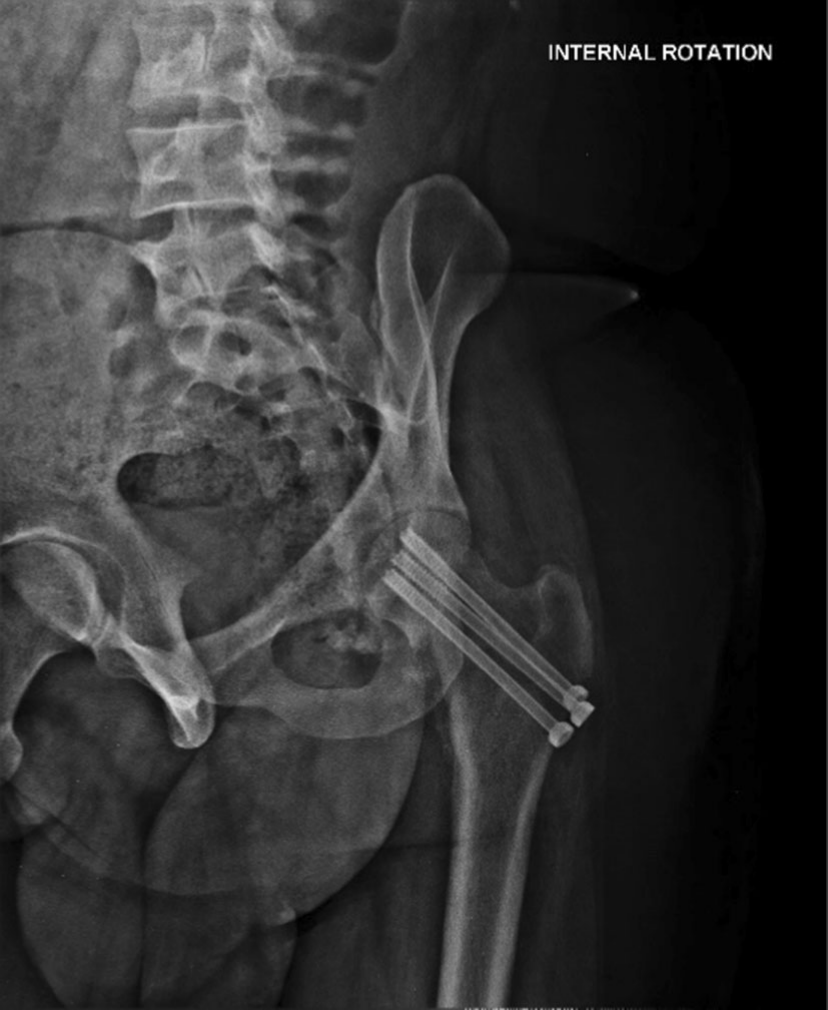
6 months postoperative X ray

X-rays 6 months after surgery showed no evidence of avascular necrosis but with a narrowing of the joint space as compared to the contralateral side. There was evidence of heterotrophic ossification around the abductor insertion (Fig. 5).

At this time, the patient was full weight-bearing with slight pain on long distance walking but no limitation in range of motion. There was a slight limp due to abductor and thigh muscles wasting. An intensified muscle-strengthening physiotherapy program was adopted with improvement in gait.

**Fig. 6:**
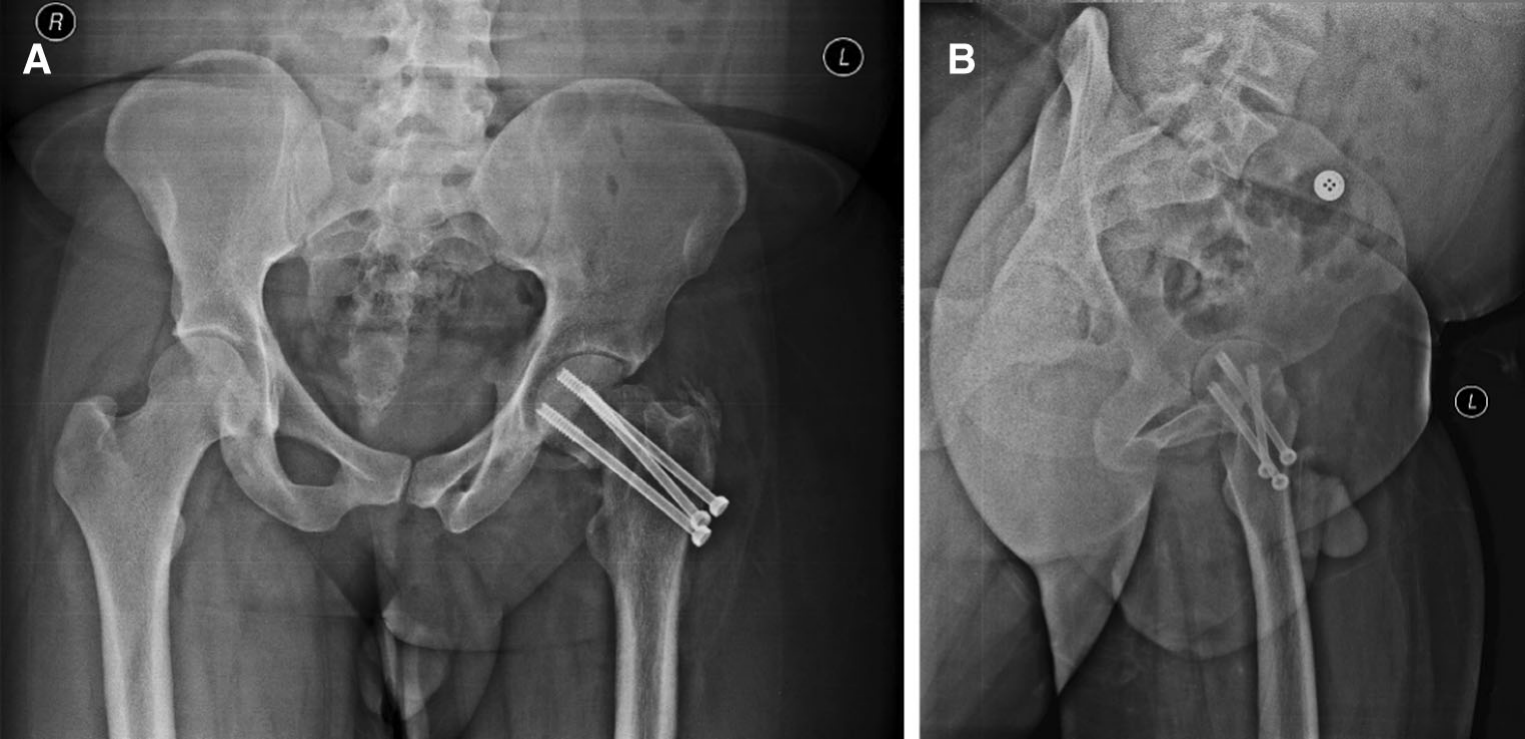
2 years postoperative X-rays: **a** Hip A/P view; showing narrowing of the joint space and heterotrophic ossification at the site of insertion of the abductors. **b** Hip lateral view

The most recent X-rays were 2 years postoperatively (Fig. 6). The final functional evaluation was carried out using the Merle d’Aubigné score as modified by Matta [10] (Table 1); this takes into account the presence of pain, ability to walk and joint range of motion. The measured functional score was 15 points out of a maximum of 18, signifying a good postoperative result.

**Table 1:** Merle d’Aubigné score modified by Matta [10]

Points	Pain	Walking	Range of motion (%)
6	None	Normal	95–100
5	Slight or intermittent	No cane, but slight limp	80–94
4	After walking, but resolves	Long distance with cane or crutch	70–79
3	Moderately severe, but patient is able to walk	Limited, even with support	60–69
2	Severe, prevents walking	Very limited	50–59
1		Unable to walk	<50

Clinical grade:

18: excellent

15–17: good

13–14: fair

<13: poor

## Discussion

A hip dislocation is an orthopedic emergency that must be reduced as soon as possible and when the patient’s condition allows. The main peculiarity of the presented case is the associated ipsilateral neck femur fracture to the anterior–inferior dislocation of the hip and the successful clinical and radiological outcome 2 years after surgery.

A posterior dislocation of the hip associated with a fracture of the femoral head [11–13] and neck [14–16] is unusual, but not rare but that of an anterior dislocation of the hip with associated fracture of the femoral head [6, 17–20] or neck [7, 14, 18, 21] much rarer still.

These injuries occur in the young active patient with 75% of cases aged <50 years and present a treatment dilemma for the surgeon [5]. A review of the literature does not provide a consensus on whether this specific injury should be treated by primary open reduction and osteosynthesis or primary hip arthroplasty [9]. Proponents of total hip arthroplasty regard it is a definitive procedure. Sadler and DiStefano reported an anterior dislocation of the hip with an ipsilateral basicervical fracture, which was reduced and fixed with a hip screw and plate. Unfortunately, their patient developed avascular necrosis, which was treated with pedicle grafting. McClelland et al. reported another case with dislocation of the obturator type with ipsilateral fractures of the femoral head and neck treated with a collarless press-fit bipolar prosthesis. Dümmer and Sanzana reported a similar case associated with subcapital fracture which was treated by an uncemented total hip arthroplasty [22]. Drummer et al. [14] treated two cases by uncemented total hip replacement, and Meller et al. [16] used an endoprosthesis to treat one case.

In contrast, many authors consider that for the young adult there is really only one treatment option which is an open reduction and internal fixation of the femoral neck fracture. Here the main goals are to preserve the femoral head, avoid osteonecrosis and avoid nonunion. Arthroplasty procedures are not ideal given the younger age and expected higher functional demands [23].

Open reduction and internal fixation are a viable option if performed within a time frame. It restores the natural bone stock, and should an arthroplasty be required subsequently for avascular necrosis or degenerative arthritis, future complications of implant loosening and revision arthroplasty are averted potentially [24].

In many cases, the radiological development of avascular necrosis may be compatible with normal activity. It takes several years before degenerative arthritis and hip pain necessitate a joint replacement. Anatomical reduction and union can help in simplifying any future arthroplasty [24]. Klasen and Binnendijk [15] treated two patients with by open reduction and internal fixation, among whom one patient had no sign of avascular necrosis at a follow-up of 8 years. Maini et al. [25] treated one patient with posterior hip dislocation and fracture of femoral neck by open reduction and internal fixation. Intraoperatively, they found no capsular attachment on the head–neck fragment. At follow-up of 2 years, despite having early changes of avascular necrosis the patient did well functionally. Primary arthroplasty is a good option for patients who are old and there is concern about nonunion; this and that of repeated surgeries potentially favor a definitive procedure at the first attempt.

There was no associated acetabulum fracture in this case. Forces acting on the femoral head of the femur put high pressure on the walls of the acetabulum, exceeding their strength, breaking them; this is a common association with hip dislocations. Finally, the absence of any other associated visceral injuries is also notable. The patient had an ipsilateral fracture clavicle and scapula. Due to high-energy trauma forces involved in producing hip dislocations, abdominal and thoracic visceral injuries, neurological or other musculoskeletal lesions can occur, but can be overshadowed by dominant hip symptoms. This emphasizes a need for a careful general examination of the patient in order accomplish a complete diagnosis.

The main complications after a hip dislocation are avascular necrosis of the femoral head leading in time to osteoarthritis. There can be heterotopic ossification around the joint and an injury of the sciatic nerve. Avascular necrosis of the femoral head is thought to be multifactorial; on the one hand, during dislocation, the vascular network emerging from the trochanteric area is damaged with the joint capsule and is the round ligament artery with this ligament. Radulescu et al. [26] mentioned that Duncan and Shim [27] demonstrated a functional disruption of cephalic circulation by a spasm of the main artery or of the cervical branches, with no organic lesion itself. Thus, early reduction in the dislocated hip decreases the risk of avascular necrosis. A delay of more than 6 h increases the risk of avascular necrosis from 10 to 40% [26].

Sendtner et al. [28] stated that osteosynthesis with preservation of the head of femur is an emergency procedure that needs to be performed within 6 h. The pressure resulting from bleeding within the articular capsule will increase after this point in time. Manninger et al. [29] reviewed 494 cases retrospectively with fracture neck femur who were treated by open reduction and internal fixation and categorized the timing of surgery into three groups: those who were operated on in the first 6 h, between 6 and 24 h and after 24 h. There was a significant difference between the first group and the other groups in the incidence of nonunion and femoral head collapse with better results in the first group and with no difference in the incidence of complications between the second and third groups.

In this patient, although there was no preserved soft tissue attachment to the femoral head, the decision for ORIF rather than primary arthroplasty was taken due to the patient’s age and occupation. Follow-up X-rays did show a narrowing of the joint space suggestive of chondrolysis together with some calcification around the insertion of the gluteal abductors. Despite this, pain was mild on long distance walking and there was no limitation in range of motion. The main postoperative complication was a limp due to abductor action and thigh muscle atrophy because of delayed weight-bearing and missed active range of motion exercises. This was much improved with physiotherapy.

## Conclusion

The clinical outcome of such cases depends on rapid assessment and management in well-equipped centers and by experienced surgical staff. In most cases, multidisciplinary emergency team is required (orthopedics, general surgery, cardiothoracic surgery, neurosurgery, radiology) together with an experienced anesthesia team with support from intensive care services. Early intervention (within 6 h) achieving stable congruent reduction and rigid internal fixation provides the best chances for such patients.

## References

[1] Mascioli AA (2017) Acute dislocations. In: Azar FM, Beaty JH, Canale ST (eds) Campbell's opertive orthopaedics, 13th edn, International edition, chapter 60. Elsevier, Philadelphia

[2] Brav EA (1962). Traumatic dislocation of the hip. J Bone Joint Surg Am.

[3] Dreinhofer KE, Schwarzkopf SR, Haas NP (1994). Isolated traumatic dislocation of the hip. J Bone Joint Surg Br.

[4] Pipkin G (1957). Treatment of grade IV fracture-dislocation of the hip a review. The Journal of Bone and Joint Surgery Am..

[5] Stewart MJ, Milford LW (1954). Fracture-dislocation of the hip. An end-result study. J Bone Joint Surg Am.

[6] Epstein HC, Harvey JP (1972). Traumatic anterior dislocation of the hip: management and results (an analysis of fifty-five cases). J Bone Joint Surg Am.

[7] Sadler AH, DiStefano M (1985). Anterior dislocation of the hip with ipsilateral basicervical fracture. A case report. J Bone Joint Surg Am.

[8] Esenkaya I, Gorgec M (2002). Traumatic anterior dislocation of the hip associated with ipsilateral femoral neck fracture: a case report. Acta Orthop Traumatol Turc.

[9] Henderson L, Johnston A, Mockford B, Craig B (2012). Posterior hip dislocation and ipsilateral isolated femoral neck fracture: a novel fixation method. J Surg Case Rep.

[10] Matta JM (1996). Fractures of the acetabulum: accuracy of reduction and results in patients managed operatively within three weeks after the injury. J Bone Joint Surg Am.

[11] Epstein HC, Wiss DA, Cozen L (1985). Posterior fracture dislocation of the hip with fractures of the femoral head. Clin Orthop.

[12] Hougaard K, Thomsen PB (1988). Traumatic posterior fracture-dislocation of the hip with fracture of the femoral head or neck, or both. J Bone Joint Surg Am.

[13] Pipkin G (1957). Treatment of grade IV fracture-dislocation of the hip. J Bone Joint Surg Am.

[14] Dümmer RE, Sanzana ES (1999). Hip dislocations associated with ipsilateral femoral neck fracture. Int Orthop.

[15] Klasen HJ, Binnendijk B (1984). Fracture of the neck of the femur associated with posterior dislocation of the hip. J Bone Joint Surg Br.

[16] Meller Y, Tennenbaum Y, Torok G (1982). Subcapital fracture of neck of the femur with complete posterior dislocation of the hip. J Trauma.

[17] DeLee JC, Evans JA, Thomas J (1980). Anterior dislocation of the hip and associated femoral-head fractures. J Bone Joint Surg Am.

[18] McClelland SJ, Bauman PA, Medley CF, Shelton ML (1987). Obturator hip dislocation with ipsilateral fractures of the femoral head and femoral neck (A case report). Clin Orthop.

[19] Richards BS, Howe DJ (1988). Anterior perineal dislocation of the hip with fracture of the femoral head (A case report). Clin Orthop.

[20] Scham SM, Fry LR (1969). Traumatic anterior dislocation of the hip with fracture of the femoral head. Clin Orthop.

[21] Hart VL (1942). Fracture-dislocation of the hip. J Bone Joint Surg.

[22] Esenkaya I, Görgeç M (2002). Dislocation of the hip and ipsilateral femoral neck fracture. Acta Orthop Traumatol Turc.

[23] Thuan VL, Swiontkowski MF (2008). Management of femoral neck fractures in young adults. Indian J Orthop.

[24] Trikha V, Goyal T, Jha RK (2011). Posterior dislocation of the hip with ipsilateral displaced femoral neck fracture. Chin J Traumatol.

[25] Maini L, Mishra P, Jain P (2004). Three part posterior fracture dislocation of the hip without fracture of the femoral head: review of literature and a case report. Injury.

[26] Radulescu R, Badila A, Japie I, Papuc A, Manolescu R, Davila C (2013). Anterior dislocation of the hip associated with intertrochanteric fracture of the femur -case presentation. J Med Life.

[27] Duncan CP, Shim SS (1977). Blood supply of the head of the femur in traumatic hip dislocation. Surg Gynecol Obstet.

[28] Sendtner E, Renkawitz T, Kramny P (2010). Fractured neck of femur—internal fixation versus arthroplasty. Dtsch Arztebl Int.

[29] Manninger J, Kazar G, Fekete G (1989). Significance of urgent (within 6 h) internal fixation in the management of fractures of the neck of the femur. Injury.

